# Multimorbidity and the risk of major adverse kidney events: findings from the UK Biobank cohort

**DOI:** 10.1093/ckj/sfab079

**Published:** 2021-04-11

**Authors:** Michael K Sullivan, Bhautesh Dinesh Jani, Jennifer S Lees, Claire E Welsh, Alex McConnachie, Bethany Stanley, Paul Welsh, Barbara I Nicholl, Donald M Lyall, Juan-Jesus Carrero, Dorothea Nitsch, Naveed Sattar, Frances S Mair, Patrick B Mark

**Affiliations:** Institute of Cardiovascular and Medical Sciences, University of Glasgow, Glasgow, UK; General Practice and Primary Care, Institute of Health and Wellbeing, University of Glasgow, Glasgow, UK; Institute of Cardiovascular and Medical Sciences, University of Glasgow, Glasgow, UK; Population Health Sciences Institute, Faculty of Medical Sciences, Newcastle University, Newcastle upon Tyne, UK; Robertson Centre for Biostatistics, Institute of Health and Wellbeing, University of Glasgow, Glasgow, UK; Robertson Centre for Biostatistics, Institute of Health and Wellbeing, University of Glasgow, Glasgow, UK; Institute of Cardiovascular and Medical Sciences, University of Glasgow, Glasgow, UK; General Practice and Primary Care, Institute of Health and Wellbeing, University of Glasgow, Glasgow, UK; Public Health, Institute of Health and Wellbeing, University of Glasgow, Glasgow, UK; Department of Medical Epidemiology and Biostatistics, Karolinska Institutet, Solna, Swedenand; Faculty of Epidemiology and Population Health, London School of Hygiene & Tropical Medicine, London, UK; Institute of Cardiovascular and Medical Sciences, University of Glasgow, Glasgow, UK; General Practice and Primary Care, Institute of Health and Wellbeing, University of Glasgow, Glasgow, UK; Institute of Cardiovascular and Medical Sciences, University of Glasgow, Glasgow, UK

**Keywords:** cardiometabolic, comorbidity, condition clusters, kidney outcomes, mortality, multimorbidity

## Abstract

**Background:**

Multimorbidity [the presence of two or more long-term conditions (LTCs)] is associated with a heightened risk of mortality, but little is known about its relationship with the risk of kidney events.

**Methods:**

Associations between multimorbidity and major adverse kidney events [MAKE: the need for long-term kidney replacement therapy, doubling of serum creatinine, fall of estimated glomerular filtration rate (eGFR) to <15 mL/min/1.73 m^2^ or 30% decline in eGFR] were studied in 68 505 participants from the UK Biobank cohort. Participants were enrolled in the study between 2006 and 2010. Associations between LTC counts and MAKE were tested using survival analyses accounting for the competing risk of death.

**Results:**

Over a median follow-up period of 12.0 years, 2963 participants had MAKE. There were associations between LTC count categories and the risk of MAKE [one LTC adjusted subhazard ratio (sHR) = 1.29, 95% confidence interval (CI) 1.15–1.45; two LTCs sHR = 1.74 (95% CI 1.55–1.96); and three or more LTCs sHR = 2.41 (95% CI 2.14–2.71)]. This finding was more pronounced when only cardiometabolic LTCs were considered [one LTC sHR = 1.58 (95% CI 1.45–1.73); two LTCs sHR = 3.17 (95% CI 2.80–3.59); and three or more LTCs sHR = 5.24 (95% CI 4.34–6.33)]. Combinations of LTCs associated with MAKE were identified. Diabetes, hypertension and coronary heart disease featured most commonly in high-risk combinations.

**Conclusions:**

Multimorbidity, and in particular cardiometabolic multimorbidity, is a risk factor for MAKE. Future research should study groups of patients who are at high risk of progressive kidney disease based on the number and type of LTCs.

## Graphical Abstract

**GRAPHICAL ABSTRACT:**
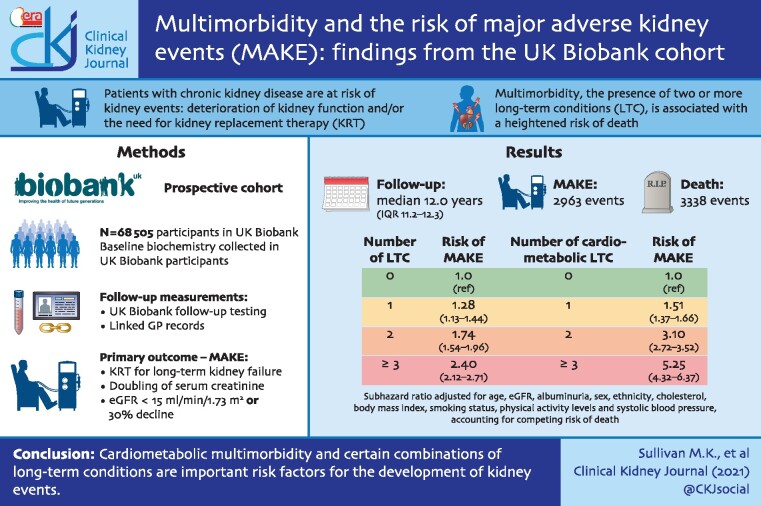

## INTRODUCTION

Multimorbidity [the presence of two or more long-term conditions (LTCs)] is a mounting problem worldwide [1, 2]. It is associated with polypharmacy [3] and increased treatment burden [4], and patients often experience poor quality of life [5]. Patients with multimorbidity are at increased risk of mortality [6, 7], and there is growing recognition that patterns of multimorbidity, or the types of LTCs, are linked to adverse outcomes [7]. Although studies have investigated the associations between multimorbidity and mortality, less is known about how the presence of multimorbidity relates to major adverse kidney events (MAKE).

With reductions in estimated glomerular filtration rate (eGFR), the risks of death, cardiovascular events and hospitalization rise [8, 9]. Tools such as the Kidney Failure Risk Equation can help predict which patients are at the highest risk of needing kidney replacement therapy (KRT) [10]. However, the risk of kidney failure is likely to be more complex than that simply defined by biochemical serum and urinary measurements [11]. Many LTCs and their treatments cause reductions in eGFR, whereas others develop as complications of chronic kidney disease (CKD) [12]. Reduced eGFR limits which tests and treatments can be used for comorbid LTCs such as the use of contrast studies for coronary angiography, and there are often conflicts between disease-specific guidelines.

Cardiometabolic LTCs (hypertension, coronary heart disease, peripheral vascular disease, atrial fibrillation, diabetes, heart failure and stroke) are particularly associated with adverse outcomes [13]. Patients with two or more cardiometabolic LTCs (cardiometabolic multimorbidity) are at high risk of death [7, 14]. Diabetes and hypertension are the two leading causes of and/or risk factors for MAKE in industrialized nations [12, 15, 16]. However, the impact of the ‘cumulative’ influence of cardiometabolic multimorbidity, rather than ‘individual’ cardiometabolic conditions, on MAKE is not well-described.

UK Biobank is a large, prospective, community-based cohort of participants with extensive phenotyping and biochemical testing. We hypothesized that in a large population study, we would observe an association between LTC counts and the future risk of MAKE. We further hypothesized that there may be specific combinations of LTCs that are associated with higher risk of developing MAKE.

## MATERIALS AND METHODS

### Study design

UK Biobank recruited 502 503 participants aged 37–73 years between 2006 and 2010. Biological data and detailed sociodemographic, lifestyle and medical information were collected at 22 assessment centres. Ethical approval was provided by the NHS National Research Ethics Service (16/NW/0274) and all participants provided written informed consent for data use and linkage of general practice (GP), hospital episode and national mortality records. This study is part of UK Biobank project 14151.

### Assessments

Blood and urine samples were collected at baseline: serum creatinine, total cholesterol and urine albumin to creatinine ratio (uACR) were measured at a centralized laboratory. The biochemistry sampling, handling and quality control protocol have been detailed previously [17]. Serum creatinine was measured using an enzymatic, isotope dilution mass spectrometry (IDMS)-traceable method on a Beckman Coulter AU5400 instrument [18] and the CKD Epidemiology Collaboration formula was used to calculate eGFR [19].

Participants self-reported their health conditions, medications and health-related behaviours at baseline. Forty-three LTCs were considered, as described in previous literature on multimorbidity in UK Biobank (see list of LTCs, Supplementary data, Table S1) [7]. All LTCs were taken from self-report other than CKD (Stages 3–5), which was defined by eGFR of <60 mL/min/1.73 m^2^ at baseline. A single blood test was used because all participants were not acutely unwell at the time of sampling. LTC counts were categorized into zero LTCs, one LTC, two LTCs and three or more LTCs. The category ‘three or more LTCs’ was chosen as the maximum category because the proportion of participants with more than four LTCs was small. Cardiometabolic LTCs were categorized in the same way.

Smoking status was divided into three categories: never, current or previous. Body mass index (BMI) was ascertained at initial assessment and used as a continuous variable. Ethnicity was coded as White, Asian, Black, Chinese, mixed or other (including Latin American). Townsend score was used to classify socio-economic status and used as a continuous variable (a higher score suggests higher levels of deprivation) [20]. The frequency of alcohol consumption was categorized as: never, special occasions only, one to three times a month, one to four times a week and daily or almost daily. Physical activity was categorized as none (no physical activity in the last 4 weeks), low (light ‘do it yourself’ activity only in the last 4 weeks), medium (heavy ‘do it yourself’ and/or walking and/or other exercises for pleasure in the last 4 weeks) and high (vigorous sports in the last 4 weeks) [21].

### Follow-up kidney function

Serum creatinine values were taken from UK Biobank follow-up testing and linked GP records. We assumed that all UK laboratories report IDMS-traceable creatinine. For individuals with more than one creatinine value, the value corresponding to the latest testing date was used. Creatinine values were identified from GP read codes (Supplementary data, Table S2) [22]. Values were excluded if the participant had an emergency admission to the hospital within 5 days of sampling, as the results would be more likely to be during a period of acute kidney injury (admissions identified from GP read codes: Supplementary data, Table S2) [23].

### Inclusion criteria

We included participants with creatinine values at baseline and at follow-up. We included participants with an eGFR of >15 mL/min/1.73 m^2^ and not receiving KRT at baseline. KRT was defined using hospital admission codes, according to a pre-specified algorithm [24].

### Study outcomes

The primary outcome was MAKE [25]: the first of the following endpoints to occur: the need to receive long-term KRT, doubling of serum creatinine, fall of eGFR to <15 mL/min/1.73 m^2^ or 30% decline in eGFR from baseline. This definition is based on previous work from the Chronic Kidney Disease Prognosis Consortium [26, 27]. All-cause mortality before MAKE was considered as a competing risk (an event that prevents the primary outcome from occurring) [28, 29]. We excluded participants who died or who had MAKE in the first 12 months of follow-up. This landmark analysis sought to exclude participants whose condition was deteriorating rapidly at recruitment [30]. The follow-up period started 12 months after the date of the first assessment and ended with the date of death, date of MAKE or end of data collection (26 April 2020), whichever occurred first.

### Statistical analysis

Demographic, physiological, prescribing and laboratory characteristics were described across LTC count categories, using medians and interquartile ranges (IQRs) for continuous variables and percentages for categorical variables. Differences in the distribution of these characteristics were tested using analysis of variance for continuous variables and Chi-squared tests for categorical variables. The characteristics of participants who had MAKE were compared with those who did not. The characteristics of participants were compared based on the availability of follow-up data: those with and without creatinine results, those with and without linked GP data, those with linked GP data with and without creatinine results, and those with and without creatinine results via UK Biobank.

Cumulative event incidence plots and Fine and Gray subdistribution hazard models were used to examine the relationship between LTC count categories and outcomes, with all-cause mortality the competing event [28, 29]. A competing risks approach was chosen over a Cox model as the preferred approach for prognostication of kidney function in the presence of a competing event such as the risk of death before MAKE [28, 31]. Participants with zero LTCs were used as the reference group. Subdistribution hazard models generated subhazard ratios (sHRs), with adjustments for confounding variables and 95% confidence intervals (CIs). Confounding variables in the standard model were age, sex, baseline eGFR, uACR, ethnicity, total cholesterol, BMI, smoking status and physical activity levels. These variables were chosen because there are associations with MAKE [12]. The proportional hazards assumption was tested using Schoenfeld residuals. Complete cases were used, which was acceptable because the proportion of participants with missing data was <5%. Analyses were repeated using cardiometabolic LTC counts. Additional analyses were performed adding adjustments for blood pressure and alcohol use. Blood pressure and alcohol use were not included in the standard model because hypertension and alcohol problems were included as self-reported LTCs. Adjustments were not made for the use of medications such as renin–angiotensin–aldosterone system blockers because of the risks of indication bias. A sensitivity analysis was performed using event plots and proportional hazard Cox models with participants censored at their date of death. These analyses were performed for total LTC counts and for cardiometabolic LTC counts with adjustments as in the standard model above.

Combinations of LTCs were identified and the associations between different LTC combinations and MAKE were studied. Competing risks models were used to identify which individual LTCs were associated with MAKE and these were used to identify all possible combinations of LTCs. To reduce the risk of multiple comparisons, we restricted our analysis to individual conditions and combinations of conditions present in >0.1% of the cohort (i.e. >68 subjects). This technique was performed for the LTC count categories two LTCs and three or more LTCs. All models were adjusted as in the standard model above. Participants with zero LTCs were used as the reference group. We reported event numbers and sHRs with 95% CIs for individual LTCs and LTC combinations associated with MAKE.

All analysis was conducted using R software version 3.6.0.

### Ethics approval

UK Biobank has full ethical approval from the NHS National Research Ethics Service (16/NW/0274).

## RESULTS

### Participant inclusion

A total of 68 505 participants met the inclusion criteria (see participant flow chart, Supplementary data, Figure S1). From the original UK Biobank cohort, 469 356 of 502 503 participants had a creatinine result at baseline. Of the 230 105 participants with linked GP data, 57 992 had one or more creatinine results during the follow-up period. A total of 16 579 participants had follow-up creatinine values recorded through UK Biobank. A total of 580 387 follow-up creatinine measurements were available. Thirty-four participants were excluded because their eGFR was <15 mL/min/1.73 m^2^ or they were on KRT at baseline. Forty-nine participants were excluded because they died or had MAKE in the first 12 months of follow-up.

### Baseline characteristics

Table 1 demonstrates the baseline characteristics of the included participants by LTC count categories. Compared with participants with zero LTCs, those with more LTCs tended to be older, female, of White ethnicity, residing in areas of greater socio-economic deprivation, smokers, with less alcohol consumption, lower physical activity levels, higher BMI, higher systolic blood pressure, higher uACR, lower total cholesterol, lower eGFR and more were prescribed antihypertensives and statins.

**Table 1.** Baseline characteristics by LTC count category

Baseline characteristic	0 LTCs, *N* = 22 348 (32.6%)	1 LTC, *N* = 22 594 (33.0%)	2 LTCs, *N* = 13 395 (19.6%)	3 or more LTCs, *N* = 10 168 (14.8%)	Total, *N* = 68 505
Age, median (IQR), years	55.0 (48.0–61.0)	58.0 (51.0–63.0)	60.0 (54.0–64.0)	61.0 (56.0–65.0)	58.0 (51.0–63.0)
Sex					
Female	11 783 (52.7)	12 072 (53.4)	7170 (53.5)	5810 (57.1)	36 835 (53.8)
Male	10 565 (47.3)	10 522 (46.6)	6225 (46.5)	4358 (42.9)	31 670 (46.2)
Ethnicity, *n* (%)					
Missing values, *n* = 217 (0.3%)					
White	21 450 (96.0)	21 860 (96.8)	13 010 (97.1)	9858 (97.0)	66 178 (96.6)
Asian	293 (1.3)	242 (1.1)	132 (1.0)	110 (1.1)	777 (1.1)
Black	162 (0.7)	155 (0.7)	71 (0.5)	53 (0.5)	441 (0.6)
Mixed	130 (0.6)	101 (0.4)	59 (0.4)	48 (0.5)	338 (0.5)
Chinese	97 (0.4)	56 (0.2)	29 (0.2)	9 (0.1)	191 (0.3)
Other	143 (0.6)	105 (0.5)	63 (0.5)	52 (0.5)	363 (0.5)
Socio-economic status based on Townsend score					
Missing values, *n* = 89 (0.1%), median (IQR)	−2.5 (−3.8, −0.2)	−2.4 (−3.8, 0.0)	−2.2 (−3.7, 0.4)	−1.7 (−3.4, 1.5)	−2.3 (−3.7, 0.2)
Frequency of alcohol consumption, *n* (%)					
Missing values, *n* = 96 (0.1%)					
Never	1246 (5.6)	1510 (6.7)	1136 (8.5)	1360 (13.4)	5252 (7.7)
Special occasions only	1921 (8.6)	2436 (10.8)	1648 (12.3)	1714 (16.9)	7719 (11.3)
One to three times a month	2388 (10.7)	2508 (11.1)	1537 (11.5)	1234 (12.1)	7667 (11.2)
Once or twice a week	6336 (28.4)	5899 (26.1)	3406 (25.4)	2390 (23.5)	18 031 (26.3)
Three or four times a week	5912 (26.5)	5531 (24.5)	2947 (22.0)	1750 (17.2)	16 140 (23.6)
Daily or almost daily	4515 (20.2)	4679 (20.7)	2706 (20.2)	1700 (16.7)	13 600 (19.9)
Physical activity (%) Missing values, *n* = 337 (0.5%)					
None	1072 (4.8)	1325 (5.9)	1069 (8.0)	1374 (13.5)	4840 (7.1)
Low	608 (2.7)	758 (3.4)	587 (4.4)	628 (6.2)	2581 (3.8)
Medium	17 314 (77.5)	18 202 (80.6)	10 782 (80.5)	7667 (75.4)	53 965 (78.8)
High	3292 (14.7)	2255 (10.0)	879 (6.6)	356 (3.5)	6782 (9.9)
Smoking status, missing values, *n* = 258 (0.4%)					
Never	13 484 (60.3)	12 656 (56.0)	6996 (52.2)	4713 (46.4)	37 849 (55.2)
Current	2180 (9.8)	2271 (10.1)	1338 (10.0)	1219 (12.0)	7008 (10.2)
Previous	6617 (29.6)	7582 (33.6)	5008 (37.4)	4181 (41.1)	23 388 (34.1)
BMI, kg/m^2^					
Missing values, *n* = 217 (0.3%), median (IQR)	25.8 (23.5–28.6)	26.7 (24.2–29.7)	27.6 (24.9–31.0)	28.9 (25.8–32.7)	26.9 (24.3–30.1)
Systolic blood pressure, mmHg					
Missing values, *n* = 3408 (5.0%), median (IQR)	134.0 (123.0–146.0)	138.0 (126.0–152.0)	140.0 (129.0–153.0)	141.0 (129.0–153.0)	138.0 (126.0–151.0)
CKD (eGFR <60 mL/min/1.73 m^2^), %	0 (0.0)	225 (1.0)	363 (2.7)	893 (8.8)	1481 (2.2)
Diabetes mellitus, %	0 (0.0)	457 (2.0)	1097 (8.2)	1870 (18.4)	3424 (5.0)
Hypertension, %	0 (0.0)	6409 (28.4)	6484 (48.4)	6572 (64.6)	19 465 (28.4)
Baseline eGFR (mL/min/1.73 m^2^), median (IQR)	94.6 (86.1–101.5)	93.0 (83.6–99.8)	91.8 (81.7–98.5)	90.1 (77.4–97.5)	92.9 (83.2–99.8)
Urine ACR, mg/mmol					
Missing values, *n* = 1612 (2.4%), median (IQR)	0.0 (0.0–0.4)	0.0 (0.0–0.6)	0.0 (0.0–0.7)	0.0 (0.0–0.9)	0.0 (0.0–0.6)
Total cholesterol, mmol/L					
Missing values *n* = 15 (0.02%), median (IQR)	5.8 (5.1–6.5)	5.7 (5.0–6.5)	5.5 (4.7–6.4)	5.3 (4.4–6.2)	5.6 (4.9–6.4)
Prescribed antihypertensives, %	221 (1.0)	5072 (22.4)	5409 (40.4)	5818 (57.2)	16 520 (24.1)
Prescribed statins, %	1012 (4.5)	3315 (14.7)	3540 (26.4)	4110 (40.4)	11 977 (17.5)

P < 0.001 for all variables (Kruskal–Wallis test for continuous variables and Chi-squared tests for categorical variables).

The participants with and without linked GP data and those with and without follow-up data from UK Biobank and GP records were similar (Supplementary data, Tables S3–S6). In those with and without follow-up data, participants had similar numbers of LTCs and the prevalence of diabetes was similar. Participants of Black and Asian ethnicities were under-represented and those with hypertension were over-represented.

### Outcomes

During a median (IQR) follow-up period of 12.0 years (11.2–12.3 years), 2963 participants had a MAKE event and 3338 died. Those with MAKE had more LTCs and more cardiometabolic LTCs (Table 2). Those with MAKE were more likely to be older, smokers, from areas of greater socio-economic deprivation, with lower consumption of alcohol, lower physical activity levels, higher BMI, higher systolic blood pressure, higher uACR, lower baseline eGFR, lower total cholesterol and proportionally more were prescribed antihypertensives and statins.

**Table 2.** Baseline characteristics by MAKE

Baseline characteristics	No MAKE, *n* = 65 542 (95.7%)	MAKE, *n* = 2963 (4.3%)
Age, median (IQR), years	58.0 (51.0–63.0)	61.0 (55.0–66.0)
Sex, *n* (%)		
Female	35 215 (53.7)	1620 (54.7)
Male	30 327 (46.3)	1343 (45.3)
Ethnicity, *n* (%)		
White	63 339 (96.6)	2839 (95.8)
Asian	727 (1.1)	50 (1.7)
Black	418 (0.6)	23 (0.8)
Mixed	326 (0.5)	12 (0.4)
Chinese	182 (0.3)	9 (0.3)
Other	342 (0.5)	21 (0.7)
Socio-economic status based on Townsend score, median (IQR)	−2.3 (−3.8, 0.1)	−2.0 (−3.6, 1.0)
Frequency of alcohol consumption, *n* (%)		
Never	4904 (7.5)	348 (11.7)
Special occasions only	7229 (11.0)	490 (16.5)
One to three times a month	7340 (11.2)	327 (11.0)
Once or twice a week	17 239 (26.3)	792 (26.7)
Three or four times a week	15 612 (23.8)	528 (17.8)
Daily or almost daily	13 129 (20.0)	471 (15.9)
Physical activity, *n* (%)		
None	4485 (6.8)	355 (12.0)
Low	2410 (3.7)	171 (5.8)
Medium	51 719 (78.9)	2246 (75.8)
High	6624 (10.1)	158 (5.3)
Smoking status, *n* (%)		
Never	36 437 (55.6)	1412 (47.7)
Current	6587 (10.1)	421 (14.2)
Previous	22 278 (34.0)	1110 (37.5)
BMI, median (IQR), kg/m^2^	26.8 (24.2–30.0)	28.3 (25.3–32.2)
Systolic blood pressure, median (IQR), mmHg	137.0 (126.0–150.0)	143.0 (131.0–157.0)
CKD (eGFR <60 mL/min/1.73 m^2^), *n* (%)	1264 (1.9)	217 (7.3)
Diabetes mellitus, *n* (%)	2817 (4.3)	607 (20.5)
Hypertension, *n* (%)	18 023 (27.5)	1442 (48.7)
LTCs, *n* (%)		
0	21 839 (33.3)	509 (17.2)
1	21 809 (33.3)	785 (26.5)
2	12 657 (19.3)	738 (24.9)
3 or more	9237 (14.1)	931 (31.4)
Cardiometabolic LTCs, *n* (%)		
0	44 439 (67.8)	1267 (42.8)
1	17 062 (26.0)	1002 (33.8)
2	3487 (5.3)	522 (17.6)
3 or more	554 (0.8)	172 (5.8)
Baseline eGFR, median (IQR), mL/min/1.73 m^2^	93.0 (83.4–99.9)	90.1 (79.6–96.6)
Urine ACR, median (IQR), mg/mmol	0.0 (0.0–0.6)	0.0 (0.0–1.5)
Total cholesterol, median (IQR), mmol/l	5.7 (4.9–6.4)	5.4 (4.5–6.2)
Antihypertensives prescribed, *n* (%)	15 154 (23.1)	1366 (46.1)
Statin prescribed, *n* (%)	10 955 (16.7)	1022 (34.5)

P < 0.001 for all variables apart from sex (0.31) and ethnicity (0.065) (Kruskal–Wallis test for continuous variables and Chi-squared tests for categorical variables).

**FIGURE 1:**
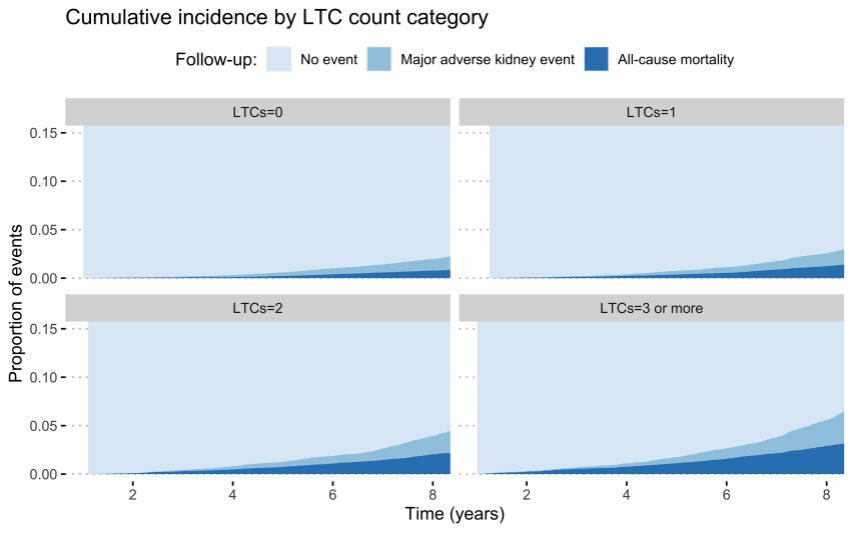
Cumulative incidence of outcomes by LTC count category.

**FIGURE 2:**
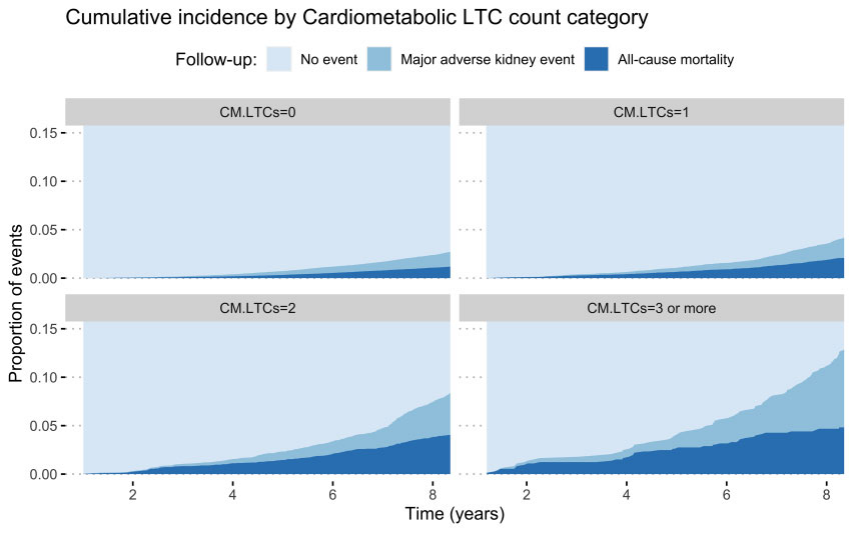
Cumulative incidence of outcomes by cardiometabolic LTC count category.

Cumulative incidences of MAKE and mortality were higher in participants with more LTCs (Figure 1). At the end of the follow-up period, 509 participants (2.3%) in the zero LTC category had MAKE, compared with 785 participants (3.5%) in the one LTC category, 738 participants (5.5%) in the two LTCs category and 931 participants (9.2%) in the three or more LTCs category. When only cardiometabolic LTCs were considered, 1267 participants (2.8%) in the zero LTC category had MAKE, compared with 1002 participants (5.5%) in the one LTC category, 522 participants (13.0%) in the two LTC category and 172 participants (23.7%) in the three or more LTC category (Figure 2).

### Competing risks analysis

The proportional hazards assumption was upheld. Associations were observed between LTC count categories and the risk of MAKE over the follow-up period (Table 3). A dose–response relationship was seen in both unadjusted and adjusted competing risks analyses. In the standard model, participants with three or more LTCs were more than twice as likely to develop MAKE than those with zero LTCs (sHR = 2.41, 95% CI 2.14–2.71). For participants with three or more cardiometabolic LTCs, the risk was more than five times greater than those with zero LTCs (standard model sHR = 5.24, 95% CI 4.34–6.33). The relationship between the number of conditions and MAKE appeared to be cumulative, whether all LTCs or just cardiometabolic LTCs were considered. Results were similar when Cox proportional hazards models were fitted, but with smaller effect sizes, including for cardiometabolic LTCs (Supplementary data, Figures S2 and S3 and Supplementary data, Tables S7 and S8). Results were similar when frequency of alcohol use was added as a covariable of interest (Supplementary data, Table S9). Most MAKE events were related to a decline of ≥30% in eGFR with 67 participants needing to initiate KRT (Supplementary data, Table S10). Competing risks analysis of each component of the MAKE definition confirmed associations between increasing LTC count category and each component, except KRT initiation (perhaps because of small event numbers). About 65.9% of those with MAKE defined from biochemical changes had blood samples that confirmed the decline in kidney function was sustained.

**Table 3.** MAKE by LTC count category: events and competing risks analysis

LTC category	*n*	MAKE, *n* (%)	Unadjusted model (sHR)	95% CI	Standard model (sHR)^a^ Missing values *n* = 2433 (3.6%)	95% CI	Additional model (sHR)^b^ Missing values *n* = 5680 (8.3%)	95% CI
LTCs								
0	22 348	509 (2.3)	1.0 (ref.)		1.0 (ref.)		1.0 (ref.)	
1	22 594	785 (3.5)	1.53	1.37–1.71	1.29	1.15–1.45	1.28	1.13–1.44
2	13 395	738 (5.5)	2.45	2.19–2.74	1.74	1.55–1.96	1.74	1.54–1.96
≥3	10 168	931 (9.2)	4.13	3.71–4.61	2.41	2.14–2.71	2.40	2.12–2.71
Cardiometabolic LTCs								
0	45 706	1267 (2.8)	1.0 (ref.)		1.0 (ref.)		1.0 (ref.)	
1	18 064	1002 (5.5)	2.02	1.86–2.20	1.58	1.45–1.73	1.51	1.37–1.66
2	4009	522 (13.0)	4.90	4.43–5.42	3.17	2.80–3.59	3.10	2.72–3.52
≥3	726	172 (23.7)	9.31	7.97–10.87	5.24	4.34–6.33	5.25	4.32–6.37

a Adjusted for age, baseline eGFR, uACR, sex, ethnicity, cholesterol, BMI, smoking status and physical activity levels.

b Adjusted for age, baseline eGFR, uACR, sex, ethnicity, cholesterol, BMI, smoking status, physical activity levels and systolic blood pressure. P-values <0.001 for all subcategories.

### Combinations of LTCs

Fourteen LTCs were present in >0.1% of the cohort and had associations with MAKE, and so were considered for potential combinations of LTCs (Table 4). For participants with two LTCs, 10 different combinations of the 14 individual LTCs were present in >0.1% of the cohort and 6 had associations with MAKE (Table 4). For participants with three or more LTCs, 29 different combinations of individual LTCs had individual associations with MAKE and 20 of these were present in >0.1% of the cohort (Table 4). For participants with two LTCs, hypertension featured in all of the combinations and for those with three or more LTCs, hypertension, diabetes, coronary heart disease and treated dyspepsia featured most commonly.

**Table 4.** LTCs and combinations of conditions that associate with MAKE

Adjusted sHRs for MAKE, when compared with reference group 0 LTCs^a^ (95% CIs)						
Individual LTCs		Category LTC count = 2, *n* = 13 395; total number with MAKE, *n* = 738		Category LTC count = 3 or more *n* = 10 168; total number with MAKE, *n* = 931		
				Index LTCs	Third LTC	
Diabetes, *n* = 607 events	3.47 (3.11–3.88)	Hypertension and CKD, *n* = 37 events	6.65 (4.26–10.38)	Hypertension, diabetes	Stroke or TIA, *n* = 36 events	11.17 (7.16–17.43)
Schizophrenia or bipolar disorder, *n* = 34 events	2.88 (2.04–4.07)	Hypertension and diabetes, *n* = 119 events	4.91 (3.72–6.48)		CKD, *n* = 51 events	9.42 (5.73–15.51)
Heart failure, *n* = 19 events	2.57 (1.53–4.32)	Hypertension and stroke or TIA, *n* = 14 events	2.89 (1.68–4.97)		Cancer, *n* = 54 events	8.24 (5.75–11.82)
Chronic liver disease, *n* = 12 events	1.90 (1.04–3.47)	Hypertension and cancer, *n* = 38 events	2.42 (1.72–3.4)		Treated dyspepsia, *n* = 60 events	8.18 (5.79–11.55)
CKD (Stages 3–5: eGFR <60 mL/min/1.73 m^2^ at baseline assessment), *n* = 217 events	1.84 (1.54–2.21)	Hypertension and coronary heart disease, *n* = 32 events	2.17 (1.47–3.19)		Psoriasis or eczema, *n* = 20 events	7.95 (4.86–13.03)
Hypertension, *n* = 1442 events	1.66 (1.53–1.79)	Hypertension and treated dyspepsia, *n* = 23 events	1.94 (1.27–2.97)		Coronary heart disease, *n* = 105 events	7.38 (5.4–10.11)
Stroke or TIA, *n* = 136 events	1.62 (1.35–1.95)				Connective tissue disease, *n* = 16 events	6.91 (3.95–12.1)
Atrial fibrillation, *n* = 45 events	1.45 (1.07–1.97)			Hypertension, CKD	Cancer, *n* = 26 events	6.28 (3.73–10.58)
Inflammatory bowel disease, *n* = 43 events	1.42 (1.01–2.01)				Coronary heart disease, *n* = 31 events	5.99 (3.57–10.05)
Coronary heart disease, *n* = 320 events	1.35 (1.18–1.54)				Stroke or TIA, *n* = 14 events	5.42 (2.67–10.99)
Cancer, *n* = 324 events	1.35 (1.20–1.53)				Treated dyspepsia, *n* = 16 events	4.64 (2.44–8.83)
Connective tissue disease, *n* = 110 events	1.30 (1.07–1.59)			Hypertension, coronary heart disease	Psoriasis or eczema, *n* = 10 events	HR 5.09 (2.63–9.87)
Psoriasis or eczema, *n* = 127 events	1.26 (1.06–1.5)				Connective tissue disease, *n* = 13 events	HR 4.74 (2.64–8.52)
Treated dyspepsia, *n* = 332 events	1.15 (1.02–1.29)				Stroke or TIA, *n* = 22 events	4.61 (2.79 –7.63)
					Treated dyspepsia, *n* = 47 events	4.58 (3.18 –6.58)
					Cancer, *n* = 22 events	3.71 (2.33 –5.92)
				Hypertension, treated dyspepsia	Connective tissue disease, *n* = 13 events	4.56 (2.57–8.09)
					Stroke or TIA, *n* = 11 events	4.26 (2.23–8.15)
					Psoriasis or eczema, *n* = 11 events	HR 3.55 (1.99–6.33)
					Cancer, *n* = 20 events	3.16 (1.98–5.06)

a Adjusted for age, baseline eGFR, uACR, sex, ethnicity, cholesterol, BMI, smoking status and physical activity levels.

TIA, transient ischaemic attack; HR, hazard ratio; CKD (Stages 3–5).

## DISCUSSION

In this study of 68 505 UK Biobank participants, we found an association between increasing LTC counts and the risk of MAKE. This finding was consistent for all LTCs, and the association with cardiometabolic multimorbidity was observed to have higher effect sizes. We identified combinations of LTCs that were associated with extremely high risk of MAKE. Diabetes and hypertension predominate in these high-risk groups, and this is not an unexpected finding. However, the substantial cumulative link and the magnitude of the association between combinations of cardiometabolic LTCs and MAKE have not been investigated in this easily understood manner before, and it is more descriptive of the clinical problem faced by clinicians caring for at-risk patients.

Our study findings are consistent with a previous study in which increasing LTC counts were associated with the need for dialysis in patients with CKD [32]. However, our approach was more comprehensive, including participants with normal and abnormal kidney function at baseline. Notably, >90% of the participants who developed MAKE did not have CKD at baseline. We have therefore shown that cardiometabolic multimorbidity is a risk factor for MAKE, even in the absence of CKD at baseline. Our definition of MAKE included a 30% fall of eGFR, which is an approach consistent with recommendations emerging from the National Kidney Foundation and the US Food and Drug Administration workshops [26, 27]. This surrogate endpoint for the development of kidney failure is important because it identifies patients before the late outcome of KRT. Studies by Bowling *et al.* [33] and Tonelli *et al.* [34] have shown that the pattern of LTCs is a risk factor in the association between multimorbidity and death in patients with CKD. Our analysis meaningfully extends this work by demonstrating that the pattern of LTCs is also linked to MAKE.

As expected, cardiometabolic LTCs and CKD were associated with MAKE. There were also associations with schizophrenia and bipolar disorder, but there were insufficient participants with these conditions for them to feature in the high-risk combinations of LTCs. It is likely that patients with mental health conditions are under-represented in UK Biobank. If a similar study was performed in the general population, high-risk groups of patients with combinations of physical and mental health problems may be identified. Some non-cardiometabolic LTCs were identified in the high-risk combinations: dyspepsia, cancer, and psoriasis or eczema. Medications used in these conditions may explain the link, or other unidentified mechanisms could be responsible. Dyspepsia has been identified in high-risk combinations of LTCs in a similar analysis studying mortality risk in patients with diabetes [35]. Although some associations with proton-pump inhibitor use and future risk of CKD have been described [36], it is unclear why these associations exist.

An important strength of our study was the inclusion of many participants with extensive phenotyping and a follow-up period that was adequate to observe the development of MAKE. The use of competing risks analysis is appropriate for studying the prognostication of kidney function, where death is a more frequent event than the kidney outcomes of interest [28].

Our study has some limitations. A large proportion of UK Biobank participants were healthy volunteers and there was under-representation of non-White and socio-economically deprived populations [37]. Analysis in a cohort with greater ethnic diversity may be necessary to confirm the generalizability of our findings to other countries. LTCs and covariates were only taken at baseline and we have not taken into account changes during follow-up because we sought to estimate the risk of progressive kidney disease from a single point in time. Our study used a select population because most of the UK Biobank cohort did not have follow-up biochemistry. Although there was a risk of selection bias (survival and ascertainment), we showed that the populations with and without follow-up biochemistry had similar characteristics. Single blood tests were used to quantify eGFR without confirmatory testing, which was deemed to be acceptable because participants were assumed to be stable at baseline assessment. Follow-up results were excluded if they were taken close to hospital admissions, but it is possible that we were unable to detect all cases of acute kidney injury. The use of self-reported LTCs is a potential limitation. However, participants were supported by a nurse in the assessment process to improve accuracy, and self-report has been found to be a valid method [38, 39].

### Potential impact

Our identification of combinations of LTCs that associate with MAKE is novel, and these high-risk groups must be studied further to identify how their risk can be reduced. Clinical leaders have highlighted that multimorbidity, rather than comorbidity, is a major global health issue and suggest that identifying clusters of conditions with clinical impacts is a research priority that could help improve the treatment of these complex patients [1, 2, 40]. Clinical guidelines should emphasize the importance of monitoring kidney function for patients with cardiometabolic multimorbidity, including those with normal kidney function. Potential interventions in these patients are intensive blood pressure and glycaemic control, lifestyle modification or planning of KRT. These interventions must always consider the priorities of patients, acknowledging their treatment burdens, which may already be significant.

Our study has demonstrated that multimorbidity, and in particular cardiometabolic multimorbidity, is a risk factor for MAKE, even in the absence of CKD. We have highlighted combinations of LTCs that are associated with high risk of MAKE in which more research is necessary to understand how risk reduction can be improved.

## Supplementary Material

sfab079_Supplementary_Data
