# Supplementary material for: Multimorbidity and the risk of major adverse kidney events: findings from the UK Biobank cohort
Source: Clin Kidney J. 2021 Apr 11;14(11):2409–19. doi: 10.1093/ckj/sfab079 (PMC8573008; doi:10.1093/ckj/sfab079)
Supplement: sfab079_Supplementary_Data [file sfab079_supplementary_data.docx]

**Supplementary Data**

| **Cardiometabolic LTCs** | **Non-Cardiometabolic LTCs** |
| --- | --- |
| Hypertension | Depression |
| Coronary Heart Disease | Painful conditions |
| Diabetes Mellitus | Asthma |
| Stroke or transient ischaemic attack | Treated Dyspepsia |
| Atrial Fibrillation | Thyroid Disorders |
| Peripheral Vascular Disease | Connective tissue disorders |
| Heart Failure | Hearing loss |
|  | Chronic Obstructive Pulmonary Disease |
|  | Anxiety & other neurotic, stress related & somatoform disorders |
|  | Irritable Bowel Syndrome |
|  | Cancer |
|  | Alcohol Problems |
|  | Psychoactive substance misuse |
|  | Treated Constipation |
|  | Chronic Kidney Disease  (eGFR < 60ml/min/1.73m^2^ at baseline assessment) |
|  | Diverticular Disease |
|  | Prostate Disorders |
|  | Glaucoma |
|  | Treated Epilepsy |
|  | Dementia |
|  | Schizophrenia or Bipolar Disorder |
|  | Psoriasis or Eczema |
|  | Inflammatory Bowel Disease |
|  | Migraine |
|  | Chronic Sinusitis |
|  | Anorexia Nervosa or Bulimia |
|  | Parkinson’s Disease |
|  | Multiple Sclerosis |
|  | Viral Hepatitis |
|  | Chronic Liver Disease |
|  | Polycystic Ovarian Syndrome |
|  | Pernicious Anaemia |
|  | Meniere’s Disease |
|  | Endometriosis |
|  | Chronic Fatigue Syndrome |
|  | Osteoporosis |

Supplementary Table S1. List of long-term conditions (LTCs) considered

| **Variable** | **Read codes** |
| --- | --- |
| GP data Creatinine Values | 44J3, 44JF, 44JD, 44JC and 4Q40 |
| GP data emergency hospital admissions | 8H2., 8H2R, 8H2F, 8H28, 8H2S, 8H24, 8H2K, h8H2, 8H2B, 8H29, 8H2, \|8H21, 8H2V, 8H230, 8H2J, 8H2O, 8H2D, 8H26, 8H2T, 8H2M, 8H2G, 8H2H, 8H2X, 8H2N, 8H2C, 8H2E, 8H2L, 8H27, 8H2A, 8Hb., 8Hd1, 8Hd5, 8Hd3 and 8Hd6 |
| ICD-10 Kidney Replacement Therapy | E85.3, N16.5, N18.0, N18.5, Q60.1, T82.4, T86.1, Y60.2, Y61.2, Y62.2, Y84.1, Z49.0, Z49.1, Z49.2, Z94.0, Z99.2, N18.0, N18.5 |
| OPCS4 Kidney Replacement Therapy | L74.1, L74.2, L74.3, L74.4, L74.5, L74.6, L74.8, L74.9, M01.2, M01.3, M01.4, M01.5, M01.8, M01.9, M02.3, M08.4, M17.2, M17.4, M17.8, M17.9, X40.1, X40.2, X40.3, X40.4, X40.5, X40.6, X40.7, X40.8, X40.9, X41.1, X41.2, X41.8, X41.9, X42.1, X42.8, X42.9, X43.1 |

Supplementary Table S2. Read codes

Supplementary Figure S1. Participant inclusion flow.

Total UK Biobank Cohort n=502,503

Participants with no baseline creatinine results n=33,147

Participants with creatinine results at baseline n=469,356

Participants with no GP follow-up data n=239,251

Participants with linked GP data n=230,105

Participants without creatinine results in GP follow-up data n=172,113

Participants with creatinine results from GP follow-up data n=57,992

Participants’ only creatinine result(s) within 5 days of a hospital admission n=210

Participants with GP creatinine results not within 5 days of a hospital admission n=57,782

Participants with creatinine results from UK Biobank follow-up n=16,579

Participants with follow-up Creatinine results available n=68,588

(5,773 participants had creatinine results from both sources)

Kidney Failure at Baseline N=34 (Estimated glomerular filtration rate <15 ml per min per 1.73 m^2^ or on kidney replacement therapy)

No Kidney Failure at baseline n=68,554

Major adverse kidney event or death in first 12 months of follow-up n=49

Included in analysis n=68,505

| **Baseline Characteristics** | | **No follow-up renal data**  N= 433705 (86.4%) | **Included participants**  N=68505 (13.6%) | **p-value** |
| --- | --- | --- | --- | --- |
| Age | Median (IQR) | 58.0 (50.0 to 63.0) | 58.0 (51.0 to 63.0) | <.001 |
| Sex (%) | Female | 236548 (54.5) | 36835 (53.8) | <.001 |
|  | Male | 197450 (45.5) | 31670 (46.2) |  |
| Ethnicity (%)  Missing = 2,776 (0.6%) | White | 406516 (93.7) | 66178 (96.6) | <.001 |
|  | Asian | 9105 (2.1) | 777 (1.1) |  |
|  | Black | 7620 (1.8) | 441 (0.6) |  |
|  | Chinese | 1383 (0.3) | 191 (0.3) |  |
|  | Mixed | 2620 (0.6) | 338 (0.5) |  |
|  | Other | 4195 (1.0) | 363 (0.5) |  |
| Socioeconomic status based on Townsend Score  Missing = 623 (0.1%) | Median (IQR) | -2.1 (-3.6 to 0.6) | -2.3 (-3.7 to 0.2) | <.001 |
| Frequency of alcohol consumption (%)  Missing = 1501 (0.3%) | Never | 35389 (8.2) | 5252 (7.7) | <.001 |
|  | Special occasions only | 50290 (11.6) | 7719 (11.3) |  |
|  | One to three times a month | 48188 (11.1) | 7667 (11.2) |  |
|  | Once or twice a week | 111260 (25.6) | 18031 (26.3) |  |
|  | Three or four times a week | 99299 (22.9) | 16140 (23.6) |  |
|  | Daily or almost daily | 88167 (20.3) | 13600 (19.9) |  |
| Physical Activity (%)  Missing = 7150 (1.4%) | None | 28008 (6.5) | 4840 (7.1) | <.001 |
|  | Low | 16357 (3.8) | 2581 (3.8) |  |
|  | Medium | 339534 (78.2) | 53965 (78.8) |  |
|  | High | 43286 (10.0) | 6782 (9.9) |  |
| Smoking status (%)  Missing = 2948 (0.6%) | Never | 235667 (54.3) | 37849 (55.3) | <.001 |
|  | Current | 45967 (10.6) | 7008 (10.2) |  |
|  | Previous | 149663 (34.5) | 23388 (34.1) |  |
| Body Mass Index (kg/m^2^)  Missing = 3105 (0.6%) | Median (IQR) | 26.7 (24.1 to 29.9) | 26.9 (24.3 to 30.1) | <.001 |
| Systolic Blood Pressure (mmHg)  Missing = 16839 (3.4%) | Median (IQR) | 136.0 (125.0 to 149.0) | 138.0 (126.0 to 151.0) | <.001 |
| Diabetes Mellitus (%) | | 22073 (5.1) | 3424 (5.0) | .331 |
| Hypertension (%) | | 113825 (26.2) | 19465 (28.4) | <.001 |
| Baseline eGFR (ml per min per 1.73 m^2^)  Missing = 33147 (6.6%) | Median (IQR) | 92.8 (82.9 to 100.0) | 92.8 (82.9 to 100.0) | .64 |
| uACR (mg mmol^−1^)  Missing = 18217 (3.6%) | Median (IQR) | 0.0 (0.0 to 0.6) | 0.0 (0.0 to 0.6) | .29 |
| Total Cholesterol (mmol l^−1^)  Missing = 32915 (6.6%) | Median (IQR) | 5.7 (4.9 to 6.4) | 5.6 (4.9 to 6.4) | .02 |
| Antihypertensives prescribed (%) | | 96984 (22.3) | 16520 (24.1) | <.001 |
| Statin prescribed (%) | | 69972 (16.1) | 11977 (17.5) | <.001 |
| LTCs (%) | 0 | 134397 (31.0) | 20229 (29.5) | <.001 |
|  | 1 | 131326 (30.3) | 21180 (30.9) |  |
|  | 2 | 83529 (19.2) | 13693 (20.0) |  |
|  | 3 or more | 84735 (19.5) | 13403 (19.6) |  |

Supplementary Table S3. Baseline characteristics by availability of follow-up data. LTCs, long-term conditions. eGFR, estimated glomerular filtration rate. uACR, urine albumin-creatinine ratio. IQR, interquartile range. P-values: Kruskal-Wallis test for continuous variables and chi-squared tests for categorical variables.

| **Baseline Characteristics** | | **GP data not linked**  N= ﻿272406 (54.2%) | **GP data linked**  N=230097 (45.8%) | **p-value** |
| --- | --- | --- | --- | --- |
| Age | Median (IQR) | 58.0 (50.0 to 63.0) | 58.0 (50.0 to 63.0) | .049 |
| Sex (%) | Female | 147600 (54.2) | 125783 (54.7) | .001 |
|  | Male | 124806 (45.8) | 104314 (45.3) |  |
| Ethnicity (%)  Missing = 2,776 (0.6%) | White | 254345 (93.4) | 218349 (94.9) | <.001 |
|  | Asian | 5174 (1.9) | 4708 (2.0) |  |
|  | Black | 5552 (2.0) | 2509 (1.1) |  |
|  | Chinese | 974 (0.4) | 600 (0.3) |  |
|  | Mixed | 1791 (0.7) | 1167 (0.5) |  |
|  | Other | 2872 (1.1) | 1686 (0.7) |  |
| LTCs (%) | 0 | 84912 (31.2) | 69714 (30.3) | <.001 |
|  | 1 | 82648 (30.3) | 69858 (30.4) |  |
|  | 2 | 52406 (19.2) | 44816 (19.5) |  |
|  | 3 or more | 52433 (19.3) | 45705 (19.9) |  |
| Socioeconomic status based on Townsend Score  Missing = 623 (0.1%) | Median (IQR) | -2.1 (-3.6 to 0.6) | -2.1 (-3.6 to 0.5) | .001 |
| Frequency of alcohol consumption (%)  Missing = 1501 (0.3%) | Never | 21738 (8.0) | 18903 (8.2) | <.001 |
|  | Special occasions only | 31782 (11.7) | 26227 (11.4) |  |
|  | One to three times a month | 29925 (11.0) | 25930 (11.3) |  |
|  | Once or twice a week | 68897 (25.3) | 60394 (26.2) |  |
|  | Three or four times a week | 62545 (23.0) | 52894 (23.0) |  |
|  | Daily or almost daily | 56596 (20.8) | 45171 (19.6) |  |
| Physical Activity (%)  Missing = 7150 (1.4%) | None | 17368 (6.4) | 15480 (6.7) | <.001 |
|  | Low | 10205 (3.7) | 8733 (3.8) |  |
|  | Medium | 212024 (77.8) | 181475 (78.9) |  |
|  | High | 27319 (10.0) | 22749 (9.9) |  |
| Smoking status (%)  Missing = 2948 (0.6%) | Never | 147780 (54.3) | 125736 (54.6) | <.001 |
|  | Current | 28789 (10.6) | 24186 (10.5) |  |
|  | Previous | 94094 (34.5) | 78957 (34.3) |  |
| Body Mass Index (kg/m^2^)  Missing = 3105 (0.6%) | Median (IQR) | 26.7 (24.1 to 29.8) | 26.8 (24.2 to 30.0) | <.001 |
| Systolic Blood Pressure (mmHg)  Missing = 16839 (3.4%) | Median (IQR) | 136.0 (125.0 to 149.0) | 137.0 (125.0 to 150.0) | <.001 |
| Diabetes Mellitus | | 13951 (5.1) | 11546 (5.0) | .096 |
| Hypertension | | 72054 (26.5) | 61236 (26.6) | .20 |
| Baseline eGFR (ml per min per 1.73 m^2^)  Missing = 33147 (6.6%) | Median (IQR) | 92.8 (82.9 to 100.0) | 92.8 (82.9 to 100.0) | .64 |
| uACR (mg mmol^−1^)  Missing = 18217 (3.6%) | Median (IQR) | 0.0 (0.0 to 0.6) | 0.0 (0.0 to 0.6) | .85 |
| Total Cholesterol (mmol l^−1^)  Missing = 32915 (6.6%) | Median (IQR) | 5.6 (4.9 to 6.4) | 5.7 (4.9 to 6.4) | <.001 |
| Prescribed Antihypertensives | | 60962 (22.4) | 52542 (22.8) | <.001 |
| Prescribed statins | | 43976 (16.1) | 37973 (16.5) | .001 |

Supplementary Table S4. Baseline characteristics by linkage of data from General Practice (GP). LTCs, long-term conditions. eGFR, estimated glomerular filtration rate. uACR, urine albumin-creatinine ratio. IQR, interquartile range. P-values: Kruskal-Wallis test for continuous variables and chi-squared tests for categorical variables.

| **Baseline Characteristics** | | **No creatinine result in linked GP data**  N= 172107 (74.8%) | **Creatinine result(s) in linked GP data**  N=57990 (25.2%) | **p-value** |
| --- | --- | --- | --- | --- |
| Age | Median (IQR) | 58.0 (50.0 to 63.0) | 58.0 (51.0 to 63.0) | <.001 |
| Sex (%) | Female | 93805 (54.5) | 31978 (55.1) | .007 |
|  | Male | 78302 (45.5) | 26012 (44.9) |  |
| Ethnicity (%)  Missing = 1078 (0.5%) | White | 162570 (94.5) | 55779 (96.2) | <.001 |
|  | Black | 2088 (1.2) | 421 (0.7) |  |
|  | Asian | 3977 (2.3) | 731 (1.3) |  |
|  | Chinese | 425 (0.2) | 175 (0.3) |  |
|  | Mixed | 857 (0.5) | 310 (0.5) |  |
|  | Other | 1352 (0.8) | 334 (0.6) |  |
| LTCs (%) | 0 | 52750 (30.6) | 16964 (29.3) | <.001 |
|  | 1 | 51984 (30.2) | 17874 (30.8) |  |
|  | 2 | 33265 (19.3) | 11551 (19.9) |  |
|  | 3 or more | 34105 (19.8) | 11600 (20.1) |  |
| Socioeconomic status based on Townsend Score  Missing = 343 (0.1%) | Median (IQR) | -2.1 (-3.6 to 0.5) | -2.2 (-3.7 to 0.5) | <.001 |
| Frequency of alcohol consumption (%)  Missing = 578 (0.3%) | Never | 14075 (8.2) | 4828 (8.3) | <.001 |
|  | Special occasions only | 19288 (11.2) | 6939 (12.0) |  |
|  | One to three times a month | 19318 (11.2) | 6612 (11.4) |  |
|  | Once or twice a week | 44941 (26.1) | 15453 (26.6) |  |
|  | Three or four times a week | 39869 (23.2) | 13025 (22.5) |  |
|  | Daily or almost daily | 34174 (19.9) | 10997 (19.0) |  |
| Physical Activity (%)  Missing = 1660 (0.7%) | None | 10801 (6.3) | 4679 (8.1) | <.001 |
|  | Low | 6378 (3.7) | 2355 (4.1) |  |
|  | Medium | 136369 (79.2) | 45106 (77.8) |  |
|  | High | 17324 (10.1) | 5425 (9.4) |  |
| Smoking status (%)  Missing = 1212 (0.5%) | Never | 94273 (54.8) | 31463 (54.3) | <.001 |
|  | Previous | 59284 (34.4) | 19673 (33.9) |  |
|  | Current | 17618 (10.2) | 6568 (11.3) |  |
| Body Mass Index (kg/m^2^)  Missing = 1406 (0.6%) | Median (IQR) | 26.8 (24.2 to 29.9) | 27.0 (24.4 to 30.3) | <.001 |
| Systolic Blood Pressure (mmHg)  Missing = 7975 (3.5%) | Median (IQR) | 137.0 (125.0 to 150.0) | 138.0 (126.0 to 151.0) | <.001 |
| Diabetes Mellitus | | 8479 (4.9) | 3067 (5.3) | .001 |
| Hypertension | | 44255 (25.7) | 16981 (29.3) | <.001 |
| Baseline eGFR (ml per min per 1.73 m^2^)  Missing = 13806 (6.0%) | Median (IQR) | 92.7 (82.7 to 100.0) | 93.1 (83.3 to 100.1) | <.001 |
| uACR (mg mmol^−1^)  Missing = 7616 (3.3%) | Median (IQR) | 0.0 (0.0 to 0.6) | 0.0 (0.0 to 0.6) | <.001 |
| Total Cholesterol (mmol l^−1^)  Missing = 13700 (6.0%) | Median (IQR) | 5.7 (4.9 to 6.4) | 5.7 (4.9 to 6.4) | .012 |
| Prescribed Antihypertensives | | 38143 (22.2) | 14399 (24.8) | <.001 |
| Prescribed statins | | 27673 (16.1) | 10300 (17.8) | <.001 |

Supplementary Table S5. Baseline characteristics by availability of creatinine results in linked General Practice (GP) data. LTCs, long-term conditions. eGFR, estimated glomerular filtration rate. uACR, urine albumin-creatinine ratio. IQR, interquartile range. P-values: Kruskal-Wallis test for continuous variables and chi-squared tests for categorical variables.

| **Baseline Characteristics** | | **No UK Biobank creatinine result available**  N=484660 (96.4%) | **UK Biobank creatinine result available**  N=17843 (3.6%) | **p-value** |
| --- | --- | --- | --- | --- |
| Age | Median (IQR) | 58.0 (50.0 to 63.0) | 59.0 (52.0 to 63.0) | <.001 |
| Sex (%) | Female | 264505 (54.6) | 8878 (49.8) | <.001 |
|  | Male | 220155 (45.4) | 8965 (50.2) |  |
| Ethnicity (%)  Missing = 2,776 (0.6%) | White | 455282 (93.9) | 17412 (97.6) | <.001 |
|  | Black | 7982 (1.6) | 79 (0.4) |  |
|  | Asian | 9761 (2.0) | 121 (0.7) |  |
|  | Chinese | 1532 (0.3) | 42 (0.2) |  |
|  | Mixed | 2894 (0.6) | 64 (0.4) |  |
|  | Other | 4476 (0.9) | 82 (0.5) |  |
| LTCs (%) | 0 | 148931 (30.7) | 5695 (31.9) | <.001 |
|  | 1 | 146920 (30.3) | 5586 (31.3) |  |
|  | 2 | 93797 (19.4) | 3425 (19.2) |  |
|  | 3 or more | 95001 (19.6) | 3137 (17.6) |  |
| Socioeconomic status based on Townsend Score  Missing = 623 (0.1%) | Median (IQR) | -2.1 (-3.6 to 0.6) | -2.7 (-4.0 to -0.8) | <.001 |
| Frequency of alcohol consumption (%)  Missing = 1501 (0.3%) | Never | 39650 (8.2) | 991 (5.6) | <.001 |
|  | Special occasions only | 56389 (11.6) | 1620 (9.1) |  |
|  | One to three times a month | 53984 (11.1) | 1871 (10.5) |  |
|  | Once or twice a week | 124775 (25.7) | 4516 (25.3) |  |
|  | Three or four times a week | 110605 (22.8) | 4834 (27.1) |  |
|  | Daily or almost daily | 97765 (20.2) | 4002 (22.4) |  |
| Physical Activity (%)  Missing = 7150 (1.4%) | None | 32147 (6.6) | 701 (3.9) | <.001 |
|  | Low | 18459 (3.8) | 479 (2.7) |  |
|  | Medium | 379341 (78.3) | 14158 (79.3) |  |
|  | High | 48006 (9.9) | 2062 (11.6) |  |
| Smoking status (%)  Missing = 2948 (0.6%) | Current | 51823 (10.7) | 1152 (6.5) | <.001 |
|  | Never | 262996 (54.3) | 10520 (59.0) |  |
|  | Previous | 166924 (34.4) | 6127 (34.3) |  |
| Body Mass Index (kg/m^2^)  Missing = 3105 (0.6%) | Median (IQR) | 26.8 (24.2 to 29.9) | 26.3 (23.8 to 29.2) | <.001 |
| Systolic Blood Pressure (mmHg)  Missing = 16839 (3.4%) | Median (IQR) | 137.0 (125.0 to 150.0) | 137.0 (125.0 to 149.0) | .61 |
| Diabetes Mellitus | | 24794 (5.1) | 703 (3.9) | <.001 |
| Hypertension | | 128999 (26.6) | 4291 (24.0) | <.001 |
| Baseline eGFR (ml per min per 1.73 m^2^)  Missing = 33147 (6.6%) | Median (IQR) | 92.8 (82.9 to 100.1) | 92.5 (83.0 to 99.0) | <.001 |
| uACR (mg mmol^−1^)  Missing = 18217 (3.6%) | Median (IQR) | 0.0 (0.0 to 0.6) | 0.0 (0.0 to 0.4) | <.001 |
| Total Cholesterol (mmol l^−1^)  Missing = 32915 (6.6%) | Median (IQR) | 5.7 (4.9 to 6.4) | 5.6 (4.9 to 6.4) | .004 |
| Prescribed Antihypertensives | | 109876 (22.7) | 3628 (20.3) | <.001 |
| Prescribed statins | | 79167 (16.3) | 2782 (15.6) | .008 |

Supplementary Table S6. Baseline characteristics by availability of creatinine results in UK Biobank data. LTCs, long-term conditions. eGFR, estimated glomerular filtration rate. uACR, urine albumin-creatinine ratio. IQR, interquartile range. P-values: Kruskal-Wallis test for continuous variables and chi-squared tests for categorical variables.


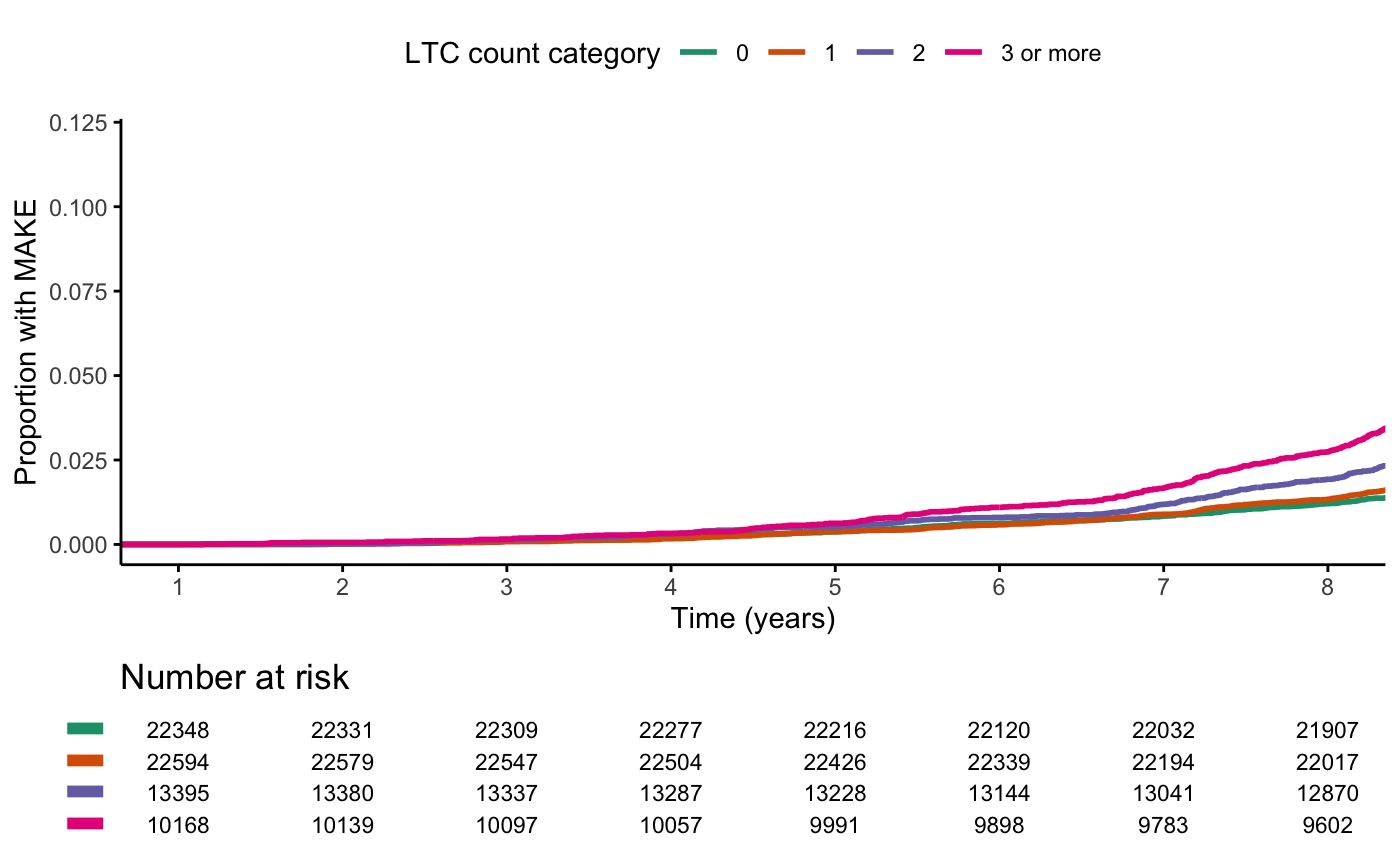


Supplementary Figure S2. Event plot for major adverse kidney events (MAKE) by long-term condition (LTC) count category

|  | | Unadjusted model (HR) | 95% CI | p-value | Standard model (HR)* | 95% CI | p-value | Additional model (HR)** | 95% CI | p-value |
| --- | --- | --- | --- | --- | --- | --- | --- | --- | --- | --- |
| LTCs | 0 | 1.0 (ref.) |  |  | 1.0 (ref.) |  |  | 1.0 (ref.) |  |  |
|  | 1 | 1.20 | 1.08 to 1.35 | .001 | 1.08 | 0.96 to 1.21 | .21 | 1.07 | 0.95 to 1.21 | .26 |
|  | 2 | 1.67 | 1.49 to 1.87 | <.001 | 1.33 | 1.18 to 1.50 | <.001 | 1.34 | 1.19 to 1.52 | <.001 |
|  | 3 or more | 2.39 | 2.15 to 2.67 | <.001 | 1.70 | 1.51 to 1.92 | <.001 | 1.71 | 1.51 to 1.93 | <.001 |

Supplementary Table S7. Proportional hazards cox regression for major adverse kidney events. *Adjusted for age, baseline eGFR, uACR, sex, ethnicity, cholesterol, BMI, smoking status & physical activity levels. **Adjusted for age, baseline eGFR, uACR, sex, ethnicity, cholesterol, BMI, smoking status, physical activity levels & systolic blood pressure.


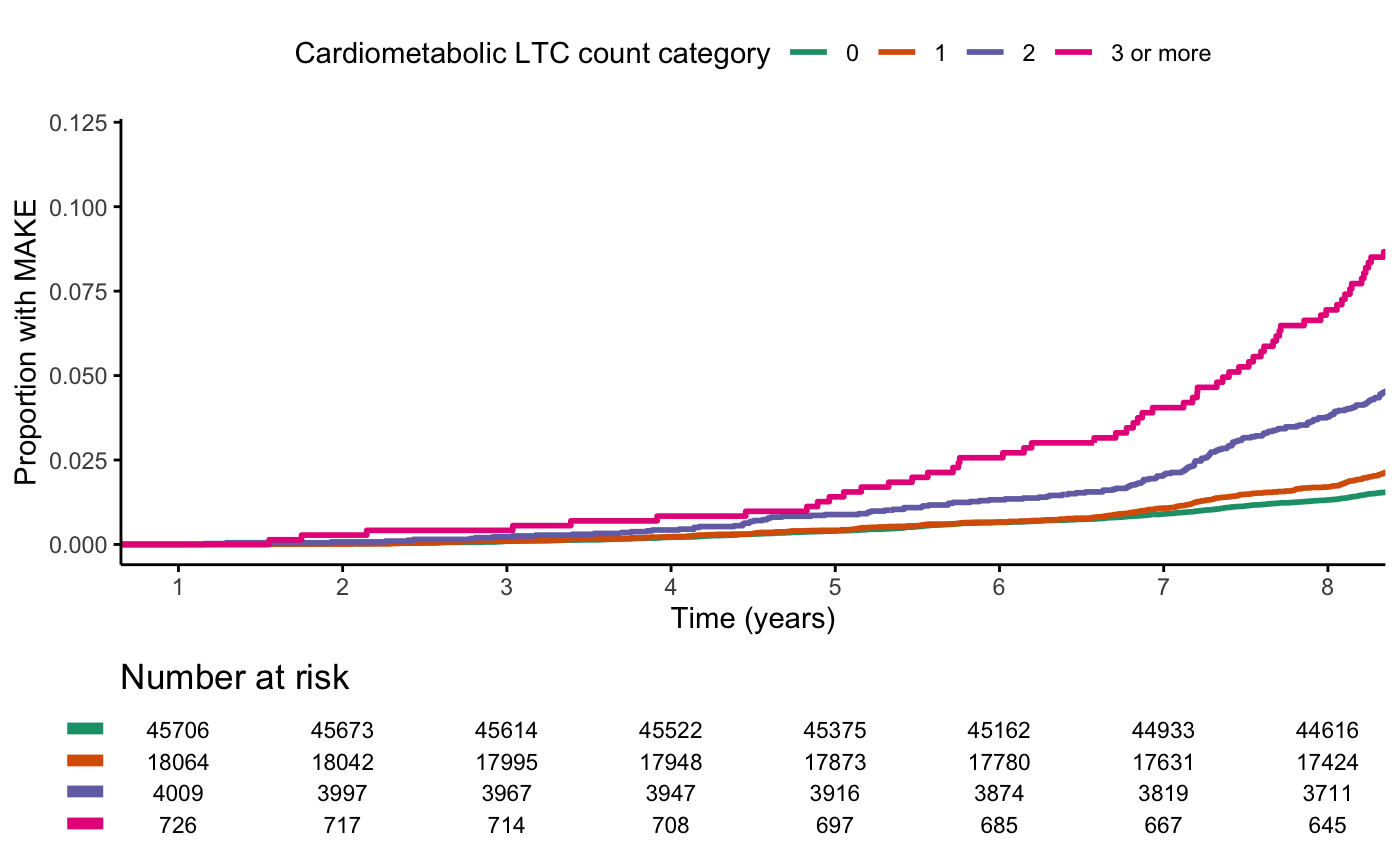
 Supplementary Figure S3. Event plot for major adverse kidney events (MAKE) by cardiometabolic long-term condition (LTC) count category

|  | | Unadjusted model (HR) | 95% CI | p-value | Standard model (HR)* | 95% CI | p-value | Additional model (HR)** | 95% CI | p-value |
| --- | --- | --- | --- | --- | --- | --- | --- | --- | --- | --- |
| LTCs | 0 | 1.0 (ref.) |  |  | 1.0 (ref.) |  |  | 1.0 (ref.) |  |  |
|  | 1 | 1.36 | 1.25 to 1.48 | <.001 | 1.16 | 1.06 to 1.27 | <.001 | 1.14 | 1.04 to 1.26 | .005 |
|  | 2 | 2.57 | 2.32 to 2.85 | <.001 | 1.98 | 1.75 to 2.23 | <.001 | 1.98 | 1.75 to 2.25 | <.001 |
|  | 3 or more | 4.18 | 3.56 to 4.91 | <.001 | 3.05 | 2.54 to 3.67 | <.001 | 3.09 | 2.56 to 3.73 | <.001 |

Supplementary Table S8. Proportional hazards cox regression for major adverse kidney events by cardiometabolic long-term condition (LTC) count category. *Adjusted for age, baseline eGFR, uACR, sex, ethnicity, cholesterol, BMI, smoking status & physical activity levels. **Adjusted for age, baseline eGFR, uACR, sex, ethnicity, cholesterol, BMI, smoking status, physical activity levels & systolic blood pressure.

| MAKE Component | LTCs | Unadjusted sHR | Adjusted sHR* |
| --- | --- | --- | --- |
| KRT initiation  N=67 | 0 | - | - |
|  | 1 | 0.99 (0.35-2.83, p=.990) | 0.81 (0.29-2.30, p=.690) |
|  | 2 | 4.56 (1.92-10.86, p=.001) | 1.63 (0.66-4.00, p=.290) |
|  | 3 or more | 9.65 (4.24-21.97, p<.001) | 2.05 (0.78-5.41, p=.150) |
| Doubling Creatinine  N=214 | 0 | - | - |
|  | 1 | 2.60 (1.46-4.63, p=.001) | 2.09 (1.17-3.74, p=.013) |
|  | 2 | 5.47 (3.12-9.58, p<.001) | 3.37 (1.92-5.93, p<.001) |
|  | 3 or more | 12.84 (7.55-21.85, p<.001) | 5.71 (3.27-9.97, p<.001) |
| eGFR fall <15ml/min/1.73m^2^  N=59 | 0 | - | - |
|  | 1 | 1.74 (0.51-5.93, p=.380) | 1.36 (0.40-4.58, p=.620) |
|  | 2 | 5.46 (1.78-16.76, p=.003) | 2.14 (0.71-6.47, p=.180) |
|  | 3 or more | 17.45 (6.16-49.45, p<.001) | 4.15 (1.38-12.44, p=.011) |
| 30% or greater fall in eGFR  N=2936 | 0 | - | - |
|  | 1 | 1.54 (1.37-1.72, p<.001) | 1.29 (1.15-1.45, p<.001) |
|  | 2 | 2.43 (2.16-2.73, p<.001) | 1.73 (1.53-1.94, p<.001) |
|  | 3 or more | 4.11 (3.68-4.59, p<.001) | 2.38 (2.12-2.69, p<.001) |

|  | | **Adjusted Subhazard Ratio*** |
| --- | --- | --- |
| LTCs | 0 | - |
|  | 1 | 1.29 (1.15-1.44, p<0.001) |
|  | 2 | 1.73 (1.53-1.94, p<0.001) |
|  | 3 or more | 2.32 (2.06-2.62, p<0.001) |
| Cardiometabolic LTCs | 0 | - |
|  | 1 | ﻿1.59 (1.45-1.74, p<0.001) |
|  | 2 | ﻿3.12 (2.76-3.53, p<0.001) |
|  | 3 or more | ﻿4.98 (4.12-6.02, p<0.001) |

Supplementary Table S9. Major adverse kidney events by long-term condition count category with added adjustment for alcohol frequency. *Adjusted for age, baseline estimated glomerular filtration rate, uACR, sex, ethnicity, cholesterol, BMI, smoking status, physical activity levels & alcohol frequency

Supplementary Table S10. Major adverse kidney events (MAKE) by long-term condition (LTC) count category: events and competing risks analysis divided by each component of the primary outcome. KRT, Kidney Replacement Therapy; eGFR, estimated glomerular filtration rate; sHR, Subhazard Ratio; *Adjusted for age, baseline eGFR, urine albumin-creatinine ratio, sex, ethnicity, cholesterol, body mass index, smoking status and physical activity levels
